# Assessing reduction in multidimensional childhood poverty in India: a decomposition analysis

**DOI:** 10.1186/s12889-023-16869-0

**Published:** 2023-10-17

**Authors:** Itishree Pradhan, Jalandhar Pradhan

**Affiliations:** Department of Humanities and Social Sciences, National Institute of Technology (NIT), Rourkela, Odisha 769008 India

**Keywords:** Multidimensional Child poverty, Alkire-Foster method, Shapley decomposition, India

## Abstract

**Background:**

Empirically, the official measurement of multidimensional poverty often shows children as the poorest age group. According to Global Multidimensional Poverty Index report, Africa and South Asia bear the highest burden multidimensional child poverty (MCP). Around one-third of children aged 0–4 are multidimensionally poor in India. Policymakers in India must have appropriate information on child poverty to alleviate poverty. The purpose of this paper is to examine MCP trends and track efforts to reduce child poverty at the national level across geographic regions, castes, and religious groups.

**Methods:**

We used the Alkire-Foster method to calculate the MCP index (MCPI) among children aged 0–4 using the latest two rounds of National Family Health Survey data (2015–16 and 2019–21). We applied the Shapley decomposition method to analyse the marginal contribution of incidence and intensity that lead to changes in MCPI.

**Results:**

In India, the incidence of child poverty reduced by more than 40% between 2015–16 and 2019–21 (46.6–27.4%) and the MCPI reduced by half (24.2–12.6%). The relative decline in MCPI has been largest for urban areas, northern regions, Other Backward Classes (OBCs) and Hindus. Children from rural areas, Scheduled Castes (SCs), Scheduled Tribes (STs), and Muslim households are the poor performers. When focusing exclusively on the poor child, we found all the population subgroups and geographic locations reduced the censored headcount ratios in all 14 indicators. Across places of residence, castes, religions, and regions the, indicators like electricity, birth registration, drinking water, assisted delivery, sanitation and cooking fuel made significant improvements between 2015–16 to 2019–21.

**Conclusion:**

The study indicates that by studying the MCPI over time, one can identify the priorities in policy development to achieve the Sustainable Development Goals.

## Introduction

Poverty entails lacking essential human requirements such as nourishment, access to safe drinking water, clothing, sanitation facilities, healthcare, shelter, education, and information [1]. Traditionally, poverty has been measured based on thresholds of unidimensional indicators such as household income or consumption, which refers all the members of a household as poor if the household does not meet the minimum defined threshold [2]. It is widely recognised that these unidimensional indicators do not capture what it means for persons of different age groups to be poor, particularly for children, because they neither have command over economic resources nor they participate in decision-making in resource allocation [3]. Sen's seminal work [4, 5] criticised the income-based definition of poverty. Income growth does not always help to alleviate deprivation in health, nutrition, safe drinking water and access to education. In this context, it's important to figure out the various aspects of poverty as well as aggregate these dimensions. Therefore, there is a pressing need to refine the multidimensional measure of poverty in order to accurately portray the range of hardships that people face across society. For children, access to primary education, good health, protection and decent housing is important for realising their basic rights and achieving their full potential for future development [6]. Hence, for the first time, the global poverty reduction goals explicitly mention children and recognise the multidimensional nature of poverty in sustainable Development Goals (SDG) target 1.2. As a result, the assessment of child poverty should account for these amenities and services. This necessitates adopting a multidimensional approach to poverty measurement, which has been explored and documented in numerous studies [7–12].

There are several reasons why multidimensional approaches are preferred over monetary approaches for measuring child poverty. First, children typically rely on their household's income but have no control over its spending; on the other hand, they depend on adults for their care, survival, growth, and development [11]. Second, numerous goods and services vital for children's development are not adequately supplied through market mechanisms. Instead, they either necessitate substantial public investment such as healthcare, education, safe drinking water, and sanitation, or they are not easily accessible in the market, such as protection from violence and exploitation, as well as participation in social activities [13]. Third, child poverty comprises more than just a lack of financial resources for the family; it also involves material and social deprivation and a dearth of basic amenities like clean water, better sanitation, healthcare, education, and information [14]. Therefore, highlighting the non-financial dimensions of child deprivation using a multidimensional index that incorporates fundamental human rights and social services is crucial for assessing child poverty [14–16]. Multidimensional child poverty (MCP) captures the diverse ways in which children experience poverty across various dimensions. This approach circumvents challenges associated with monetary measures of poverty, such as the complexities of intra-household resource allocations [13, 17].

According to the Global Multidimensional Poverty Index (MPI), 2022, nearly 20 percent (1.2 billion) of people who live in the 111 countries studied were living in multidimensional poverty, and about half (593 million) of them are children under the age 18 [18]. The study further found that one in three children is multidimensionally poor compared with one in six adults [18], indicating that child poverty is becoming a significant global concern. Two out of five (44%) multidimensionally poor children live in South Asia, where half of the children experience multidimensional poverty [19]. More than half of India's multidimensionally poor are children aged 0–17 years [19]. In addition to the above disquieting statistics of MCP, it is well documented that living in poverty as a child has long-term repercussions that negatively impact adult life prospects and society as a whole. Besides its lifelong consequences, it differs from adult poverty [3, 6, 20–23]. Therefore, combating child poverty should be a top priority to ensure everyone has access to equitable chances [15]. Additionally, considering children as a distinct population subgroup enables deeper attention to the details of children's specific needs. Numerous studies on MCP have been carried out in both developed and developing nations for different age groups of children [2, 8, 10, 15, 19, 20]. However, very little has been documented about MCP in the context of India, even though nearly half of the Indian population is under 18 years [9, 13, 23–25].

In terms of its growth trajectory, India has made significant progress in various sectors [26]. The growth rate of India's GDP has been stable since the mid-1990s, with an annual average of 6 to 7% [27]. It is critical to ensure that the advantages of growth reach all segments of society. The analysis of poverty trends is necessary to determine whether or not the growth performance is inclusive. It is critical to determine whether it has worsened regional disparities, the rural–urban divide, and socioeconomic inequality [26]. Various researchers show the trends of MCP in their studies across different countries such as in Bangladesh, China, Ethiopia, Ghana, India, Ireland, Peru, and Vietnam, and OECD countries [14, 28–36].

As an interesting triangulation, Mishra & Ray [37] found a smaller decline in MPI among India's most impoverished castes (Scheduled Caste (SCs) and Scheduled Tribes (STs)). Alkire & Seth [38] observed India's MPI decreased, but the poorest subgroups progressed more slowly, extending the intergroup disparity. Alkire et al. [39] found that the poorest of the poor saw the largest reductions in MPI during 2005–06 to 2015–16. Das et al. [40] demonstrated over the past decade (2005–2006 and 2015–2016), MPI has decreased across regions of India. Again Das et al. [41] noted the reduction of multidimensional poverty was faster across the poorer groups in India. Similarly, Das et al. [26] pointed out that in India, there has been a large reduction in MPI between 2015–16 to 2019–21, and the worst performing states in 2005–06 also performed worst in 2019–21. Based on the aforementioned context, we can conclude that there is a scarcity of impartial research on multidimensional poverty patterns in India. And in multidimensional poverty analysis, all the above pieces of literature have considered the household as the unit of analysis and count all the members of a poor household as multidimensionally poor. But this study is based on child specific analysis. Furthermore, to the best of the authors' knowledge, no research has analysed recent changes in MCP in India. These assessments are especially important for assessing the events in MCP and India's concern for SDGs.

There is a dearth of literature on MCP trends in the Indian context. Against this backdrop, the aim of this study is to examine changes in multidimensional child poverty in India from 2015–16 to 2019–2021. The contribution of this research differs from previous works in that it measures recent trends in multidimensional child poverty for India utilising dense data sets from two recent waves of the National Family Health Survey (NFHS) from 2015–16 to 2019–21. The selection of dimensions, indices, and weights for measuring MCP is crucial. This study examines changes in multidimensional child poverty at the national level and trends at the disaggregate level, taking into account the rural–urban classification, social group classification, religious group classification and region-wise classification. Analyses in this field aid in formulating various policies, particularly those targeting vulnerable social groups, religious communities, and geographical areas.

## Data and method

For the estimation and analysis of MCP in India across places of residence, castes, religions and geographic regions, we used unit-level data from the most recent two rounds, NFHS-4 (2015–16) and NFHS-5 (2019–21), conducted by the International Institute of Population Sciences (IIPS), Mumbai, under the Ministry of Health and Family Welfare, Government of India [42]. Both NFHS datasets are nationally representative, covering 29 states and seven union territories. Samples for both surveys are drawn using a multi-stage stratified sampling technique based on the 2011 Census. As a result, the two data sets are comparable overtime at the state and district levels. The sample designs for the two surveys are substantially identical, with a few variations. The NFHS-5 covered a nationally representative sample of 2,843,917 individuals across 636,699 households, and the NFHS-4 covered sample of 2,869,043 individuals across 601,509 households. This study has collected precise data on health, nutritional status, mortality, sociodemographic characteristics, access to basic facilities, and household assets, all required for MCP calculations. In NFHS-4, 259,374 children aged 0–4 years are retained for the analysis. A total of 234,588 children were retained from NFHS-5 for the analysis. This study excluded children who were deceased, had missing information about any of the 14 indicators, or were not usual household residents. The dead children were dropped from the sample because they did not have some information such as anthropometric measurements and immunisation; moreover, they cannot be referred to as someone who is experiencing or living in poverty. The non-usual resident children are visitors to the household. Hence all household-specific information pertinent to children does not apply to them, and locating their actual household is not possible in the NFHS data. This sample selection procedure is based on the various existing studies on multidimensional poverty analysis [18, 39, 43, 44].

### Methodology

In this study, we have employed the Alkire-Foster (AF) counting methodology [43] to determine the MCP among geographic location and population subgroups in India. The A-F technique's proposed conceptual framework is based on Amartya Sen's 'capability approach' [43]. Sen's capability approach provides a useful framework for building a multidimensional framework for child poverty. In the context of child poverty, the capability approach would consider children's capabilities and opportunities for development and well-being. The dimensions of health, education, early childhood development, protection, and material deprivation would be seen as capabilities that children should have access to in order to realise their full potential and lead fulfilling lives [44, 45]. Based on the A-F methodology, MCPI is constructed using two analytical steps: identification and aggregation. In a dual cutoff identification technique, deprivation and poverty cutoffs are both employed to identify poor children. A deprivation cutoff is employed for each indicator to determine whether a child is deprived in that particular indicator, and each child's final deprivation score is obtained by summing up their weighted deprivation score of all the indicators. Detailed information on the deprivation cutoff and indicator weights are shown in Table 1. The following sections discuss the selection of dimensions and indicators and the process of calculating the deprivation score.

**Table 1:** Dimensions, indicators, deprivation cutoffs, and indicators' weight

Dimension	Indicators	Deprived if / Deprivation Cutoff	Weight
**Health**	Stunting	Child's Height-for-Age below -2 standard deviation from WHO reference	1/16
	Wasting	Child's Weight-for-Height below -2 standard deviation from WHO reference	1/16
	Assisted Delivery	Child's birth is not assisted by any health personnel^a^	1/16
	Full Immunisation	Child is not timely vaccinated with all age-appropriate vaccines^b^	1/16
**Early Childhood Development**	Mother's Education	Mother has no formal schooling	1/8
	Birth Registration	Child's birth is not registered	1/8
**Standard of Living**	Housing Condition	Child lives in an inadequate housing condition i.e., floor/roof/wall is made of natural/rudimentary materials^c^	1/20
	Electricity	No Electricity at home	1/20
	Cooking Fuel	Household uses unclean/unimproved cooking fuel^d^	1/20
	Overcrowding	Household has more than 4 members per sleeping room (rooms excluding kitchen)	1/20
	Information	Household does not have access to Television/Radio/Newspaper	1/20
**WASH**	Drinking Water	Household does not have access to safe drinking water^e^, or the round-trip walking time from the house to the source is more than 30 min	1/12
	Sanitation Facility	Household does not have an improved toilet facility^f^ or the facility is shared with other households	1/12
	Hand Hygiene	Household does not have a specific place for hand hygiene, or water and soap or other cleansing agent^g^ is not present at the place for hand hygiene	1/12

Source: The indicators were derived from India's National Multidimensional Poverty Index Baseline Report [46] & SDGs Targets [47]

^a^Health personnel: "Doctor, Auxiliary Nurse Midwives (ANM), Nurses, Midwives & Lady health visitors"

^b^Age-appropriate Vaccines: 0–5 Months → BCG, OPV-0, and Hep B-0; 6–11 Months → BCG, OPV-0, Hep B-0, OPV-1, 2, 3, and Pentavalent- 1, 2, 3; 12–23 Months → BCG, OPV-0, Hep B-0, OPV-1, 2, 3, Pentavalent- 1, 2, 3, and MMR/Measles-1; 24–35 Months → BCG, OPV-0, Hep B-0, OPV-1, 2, 3, Pentavalent- 1, 2, 3, MMR/Measles-1, 2, and DTP Booster-1. Where BCG-Bacillus Calmette-Guerin; Hep B-Hepatitis B; OPV-Oral Polio Vaccine; DTP-Diphtheria, Tetanus, and Pertussis; MMR-Measles, Mumps, and Rubella vaccine; and the numbers indicate a dose order

^c^Floor: mud/earth, sand, dung, other; Wall: no wall, cane/palms/trunk, mud/dirt; Roof: no roof, thatch/palm leaf mud/earth/lump of earth

^d^Unclean cooking fuels: “Coal/Lignite; Charcoal; Wood; Straw/shrubs/grass; Agricultural crop; Animal dung”

^e^Source of Safe Drinking Water: "Piped water into dwelling/yard/plot; Public tap/Standpipe; Tube well or Borehole; Protected dug well; Protected spring; Rainwater; and community RO plant"

^f^Improved toilet facility: "Flush/pour flush to piped sewer system, septic tank, or pit latrine; Pit latrine with slab; Ventilated improved pit (VIP)/biogas latrine and twin pit/composting toilet"

^g^Cleaning agents other than soap include locally available materials such as ash, mud or sand

### Selection of dimensions and indicators

The United Nations (UN) has restated that investing in children and ensuring the fulfilment of their rights are highly impactful strategies for eliminating poverty [48]. The UN further recommends that poverty analysis should encompass all fundamental human rights and necessities, including access to nutritious food, safe drinking water, adequate sanitation, proper healthcare facilities, shelter, and education [47]. This justifies the selection of the dimensions and indicators for this study, and violations of these basic rights are treated as deprivations. Based on these human needs and necessities, in this study, MCPI was calculated using data from 14 indicators across four key dimensions: Health, Early Childhood Development (ECD), Standard of Living, and Water, Sanitation and Hygiene (WASH), all of which were given equal weightage for children aged 0–4 years. Earlier studies have also recognised these dimensions /indicators for measuring multidimensional child poverty [2, 7, 13, 19, 20, 44, 49, 50]. The detailed explanation on the selected dimensions and indicators can be found in our previous study [50]. Detailed information on the dimension, indicator, poverty cutoff and indicator's weight are shown in Table 1.

### Computation of Multidimensional Child Poverty Index (MCPI)

Unlike the three key deprivation dimensions —Health, Education, and Standard of living— used by Alkire & Foster [43], a total of four dimensions comprising 14 indicators for 0–4 years of children were included in this study for measuring the MCPI. Each of the four dimensions was given an equal weight of 1/4 since they are considered equally important for a child's optimal growth and development for 0–4 years children. Then, the weights of the indicators are assigned by dividing the dimensional weight (1/4) into equal portions according to the number of indicators in the dimensions. Since there are different numbers of indicators under each dimension, the indicators' weight was unevenly assigned. Based on the criteria in Table 1 for determining whether or not a child is deprived, a score of "1" will be assigned to the child if she/he is deprived in that indicator and a score of "0" otherwise. Then, the overall deprivation score is calculated as the weighted sum of the score obtained in all 14 indicators. Next, a poverty cutoff evaluates whether a person falls into the multidimensionally poor category. If a person's deprivation score is more than or equal to that poverty cutoff, they are considered multidimensionally poor. Here the poverty cutoff is denoted as 'k', and following the global MPI cutoff of '1/3', the cutoff point for being multidimensionally poor is set at k = 0.333. There is no specific criterion for determining the multidimensional poverty threshold [18, 51]. Therefore, in the absence of a universally accepted standard, we rely on the experiences and findings of various studies to set the multidimensional poverty threshold. The cutoff of 0.333 (33.33%) has been used in a number of studies to measure multidimensional child poverty, irrespective of the number of dimensions considered [13, 22, 52, 53]. Here the poverty cutoff is denoted as 'k', and following the previous literature on MCP the cutoff point for being multidimensionally poor is set at k = 0.333. Suppose the 'q' be the total number of individuals whose overall deprivation score is ≥ k = 0.333, and the total sample size of the study is 'n'; the traditional headcount ratio or incidence (H) is computed as in Eq. (1):

1 $$H=\frac{q}{n}$$

While the H indicates the proportion of multidimensionally poor child in the total child population, the uncensored headcount ratio (denoted by h_j_) indicates the proportion of child who are deprived in an indicator' j' regardless of whether they are multidimensionally poor or not. The formula is as follows: $h_i=\frac1n{\textstyle\sum_{i=1}^ng_{ij}^o}$

Where $\sum_{i=1}^{n}{g}_{ij}^{0}$ is the sum of the deprivation status for the indicator' j' up to the 'i^th^' individual and 'n' is the total child population. The uncensored headcount ratios have been reported as percentages (h_j_*100) in this study.

Similar to its uncensored counterpart, the censored headcount ratio (denoted by h_j_(k)) indicates the proportion of children who are multidimensionally poor and deprived in an indicator' j'. It is computed as ${h}_{j}\left(k\right)=\frac{1}{n}\sum_{i=1}^{n}{g}_{ij}^{0}(k)$

Where 'n' is the total population, and ${g}_{ij}^{0}(k)$ is the censored deprivation score of individual' i' in the indicator 'j' using a second-order cutoff (k) of 33.33 percent. In this study, it has been reported as percentages (h_j_(k)*100).

Nonetheless, the conventional approach fails to provide an accurate representation of the extent to which individuals are profoundly impacted by multidimensional poverty. Additionally, if impoverished individuals experience further deprivation in different aspects, this approach does not account for the increased severity [54]. To overcome this, the AF method was introduced, aiming to enhance the measurement by introducing the concept of intensity in multidimensional poverty (A). This intensity signifies the average deprivation score among those experiencing multidimensional poverty and is formulated as follows:

2 $$A=\frac{C(k)}{q}=\frac{\sum_{i}^{q}{C}_{i}(k)}{q}$$

In this context, '${C}_{i}(k)$' represents the deprivation score of an individual '$i$' experiencing multidimensional poverty, while '$q$' refers to the total number of people identified as multidimensionally poor.

Consequently, the AF method proposes the MPI (in this study, it is the MCPI) or the adjusted headcount ratio, ${M}_{0}$ By multiplying the H with the A, represented in the equation below:

3 $$MCPI={M}_{0}=H\times A$$

Additionally, M_0_ meets the axioms of Ordinality, dimensionality breakdown, and population subgroup decomposability. Dimensional Breakdown connects multidimensional child poverty levels to dimensional components, whereas Subgroup Decomposability and Ordinality allow for valid assessments of poverty when variables are ordinal. This study aimed to facilitate comparisons among Castes, religions, places of residence and regions by decomposing M_0_ according to them.

#### Changes in M0, H and A across two time periods

This section explains how to use repeated cross-sectional data to compare the MCPI and its associated partial indices (H and A) over time. Migration and demographic shifts may also have a significant impact on such comparisons, necessitating independent treatment [39]. Poverty comparisons rely on the core concept of measuring absolute changes over time, as highlighted by Alkire & Seth [38]. Changes in poverty (increases or decreases) over two time periods can also be reported as a relative rate. These changes, whether increases or decreases, can also be presented as relative rates. The absolute rate of change simply refers to the numerical difference between two time periods, while the relative rate of change is the difference between two periods expressed as a percentage of the initial period. By combining both absolute and relative changes, we can gain a basic understanding of overall progress, as emphasised by [55]. In this context, the initial period is denoted as ${t}^{1}$, and the final period is denoted as ${t}^{2}$. ${Y}_{{t}^{1}}$ and ${Y}_{{t}^{2}}$ represent the achievement matrices for periods ${t}^{1}$ and ${t}^{2}$, respectively. However, for the sake of clarity, we provide M_0_ and its partial indices solely as a function of the achievement matrix. To ensure strict comparability across time, it is essential to employ the same set of parameters for both periods.

The absolute rate of change, denoted as Δ, in Adjusted Headcount Ratios between two time periods can be calculated as follows:$\Delta {\mathrm{M}}_{0}={\mathrm{M}}_{0} ({Y}_{{t}^{2}})-{\mathrm{M}}_{0} ({Y}_{{t}^{1}})$

Similarly, for the partial indices H and A, the absolute rate of change is computed as follows: $\Delta \mathrm{H }=\mathrm{ H }\left({Y}_{{t}^{2}}\right)-\mathrm{H}({Y}_{{t}^{1}})$ and $\mathrm{\Delta A}=\mathrm{A}\left({Y}_{{t}^{2}}\right)-\mathrm{A}({Y}_{{t}^{1}})$

On the other hand, the relative rate of change represented by δ, in Adjusted Headcount Ratios between two time periods can be calculated as follows: $\mathrm{\delta M}0 =\frac{\mathrm{M}0 \left({Y}_{{t}^{2}}\right)-\mathrm{M}0 ({Y}_{{t}^{1}}))}{\mathrm{M}0 ({Y}_{{t}^{1}})}*100$

The same formulas mentioned above can also apply to each partial indices, namely H and A, uncensored headcount ratio, censored headcount ratios, or percent contributions.

### Shapley decomposition of the change in MCP by subgroups:

The Alkire-Foster method does not allow us to estimate the contribution of each subgroup to the changes in poverty [32]. Whereas the Shapley decomposition method allow us to estimate how much has each subgroup contributed to overall reduction in child poverty. Shapley decomposition is used to analyse the distributional impact of changes in poverty across subgroups. Here we follow the disaggregation method suggested by Roche (2013) [32]. The differences in poverty levels can be separated into changes resulting from effects within specific sectors or groups (intra-sectoral or within-group poverty effects) and changes resulting from demographic or inter-sectoral effects via:

4 $${\mathrm{\Delta M}}_{0}={\sum }_{i=1}^{m}\left(\frac{{P}_{i}^{{t}^{2}}+{P}_{i}^{{t}^{1}}}{2}\right)\left({M}_{0i }^{{t}^{1}}-{M}_{0i}^{{t}^{2}}\right) + {\sum }_{i=1}^{m}\left(\frac{{M}_{oi}^{{t}^{2}}+{M}^{{t}^{1}}}{2}\right)\left({P}_{i}^{{t}^{1}}-{P}_{i}^{{t}^{2}}\right)$$

The term ΔM_0_ represents the overall change in the adjusted headcount ratio between two time periods, ${t}^{1}$ (2015–16) and ${t}^{2}$ (2019–21). It is calculated as the difference between the adjusted headcount ratio of subgroup ‘i’ at time ${t}^{1}$, i.e., ${M}_{0i }^{{t}^{1}}$ and the adjusted headcount ratio subgroup i at the time ${t}^{2}$, i.e., ${M}_{0i }^{{t}^{2}}$. In addition, ${P}_{i}^{{t}^{1}}$ represents the population share of subgroup i out of a total of m subgroups (i = 1,………m) in the respective time periods. It is important to note that the Shapley decomposition allows for estimating the individual contributions of the within-group effect and the demographic effect.

The Shapley decomposition method allows for analysing changes in poverty based on its incidence and intensity. The adjusted headcount ratio, represented as (Mo = H * A), reflects the combined impact of multidimensional poverty incidence and intensity among poor children. By applying the Shapley decomposition technique developed by Shorrocks in 2013 [56], we can break down the absolute change in the adjusted headcount ratio into two distinct effects: the incidence and the intensity effect. That is

5 $${\mathrm{\Delta M}}_{0}= \frac{{A}^{{t}^{2}}+{A}^{{t}^{1}}}{2} ({H}^{{t}^{1} }-{H}^{{t}^{2}})+ \frac{{H}^{{t}^{2}}+{H}^{{t}^{1}}}{2} \left({A}^{{t}^{1} }-{A}^{{t}^{2}}\right)$$

In the given Eq. (5), ${H}^{{t}^{1}}$ and ${A}^{{t}^{1}}$ represent the headcount ratio and the intensity of poverty in the time period ${t}^{1}$ (2015–16), while ${H}^{{t}^{2}}$ and ${A}^{{t}^{2}}$ represent the headcount ratio and the intensity of poverty during the time period ${t}^{2}$ (2019–21) respectively. The first part of Eq. (5) represents the effect of poverty incidence, indicating how changes in the headcount ratio contribute to overall poverty change. The second part of the equation represents the effect of poverty intensity, showing how changes in the severity or depth of poverty contribute to overall poverty change.

The Alkire-Foster intensity of poverty, denoted as A, represents the average deprivation share among the poor individuals. It can be calculated as $A=\frac{C(k)}{q}=\frac{\sum_{i}^{q}{C}_{i}(k)}{q}$ where c(k) represents censored number of weighted deprivations for individual ‘i’ and q is the number of individuals identified as multidimensionally poor. To understand changes in intensity of poverty, it is possible to decompose it, based on the changes in deprivations experienced by the poor in each specific dimension or indicator. We know that intensity at time t, can be expressed as A^t^ = $\sum_{j=1}^{d}\left({w}_{j }{h}_{j}^{t}\right)/d$. Here ${w}_{j}$ signifies the dimensional or indicator weight, with $\sum_{j=1}^{d}{w}_{j }=d$ (i.e. the sum of all dimensional weights is equal to ‘d’). Additionally, ${h}_{j}^{t}$ represents the share of the poor who are deprived in dimension j at time t.

The decomposition of the absolute change in intensity is conducted as follows when the dimensional weight remains constant across the period:

6 $$\mathrm\Delta\mathrm A=\sum\nolimits_{j=1}^d\left(w_j/d\right)\left(h_j^2-h_j^1\right)$$

It is worth noting that ${h}_{j}^{t}$ can alternatively be represented as ${h}_{j}^{t}$= ${}^{{CH}_{j}^{t}}\!\left/ \!{}_{{H}^{t}}\right.$, where ${CH}_{j}^{t}$ denotes the censored headcount ratio of dimension j at the time ‘t’, and ${H}^{t}$ represents the proportion of poor children n/q. Thus, Eq. (6) can be conveniently expressed as a function of the censored headcount ratio in each dimension, allowing for a more straightforward analysis of the data, i.e.

7 $$\mathrm{\Delta A }= \sum\nolimits_{j=1}^{d}\left({}^{{w}_{j}}\!\left/ \!{}_{d}\right.\right) \left({}^{{CH}_{j}^{2}}\!\left/ \!{}_{{H}^{2} }\right.-{}^{{CH}_{j}^{1}}\!\left/ \!{}_{{H}^{1}}\right.\right)$$

Conducting an integrated analysis can bring significant advantages and provide a more holistic picture by combining the decomposition of poverty changes by subgroup (Eq. 4), with decomposition by its components (Eq. 5), and decomposition by dimensions or indicators (Eq. 7) as follows:

8 $${\mathrm{\Delta M}}_{0}= \sum\nolimits_{i=1}^{m}\left(\frac{{M}_{0i }^{{t}^{2}}+{M}_{0i }^{{t}^{1}}}{2}\right)\left({P}_{i}^{{t}^{1}}-{P}_{i}^{{t}^{2}}\right) + \sum\nolimits_{i=1}^{m}\left(\frac{{P}_{i}^{{t}^{2}}+{P}_{i}^{{t}^{1}}}{2}\right) (\frac{{A}^{{t}^{2}}+{A}^{{t}^{1}}}{2}) ({H}^{{t}^{1} }-{H}^{{t}^{2}}) + \sum\nolimits_{i=1}^{m}\left(\frac{{P}_{i}^{{t}^{2}}+{P}_{i}^{{t}^{1}}}{2}\right) (\frac{{H}^{{t}^{2}}+{H}^{{t}^{1}}}{2}) \sum\nolimits_{j=1}^{d}\left(\frac{{w}_{j}}{d}\right) \left(\frac{{CH}_{J}^{2}}{{H}^{2}}-\frac{{CH}_{J}^{1}}{{H}^{1}}\right)$$

## Results

### Changes in H, A and MCPI across the geographic regions and population subgroups

We examined the changes in multidimensional child poverty for children aged 0–4 years between the periods 2015–16 and 2019–21. We then investigated the specific population subgroups where the reduction has occurred. Table 2 provides information on MCPI and its two components, ‘H’ and ‘A’, using a poverty cut-off of k = 1/3. Table 2 indicates, India has made a momentous achievement because the national MCPI has halved between 2015–16 and 2019–21, i.e., from 0.241 to 0.126. There was also an absolute reduction in ‘H’ and ‘A’, but the magnitude of the reduction of H was more prominent. The incidence of poverty reduced strongly, i.e., from 46.6% in 2015–16 to 27.4% in 2019–21, i.e., the percentage of multidimensionally poor children (H) declined by 19.2 percentage points. However, the reduction of MCP intensity was relatively low in India. It is crucial to investigate the changes of poverty within various subgroups. Furthermore, it should also be noted that significant reductions in H are not necessarily accompanied by equally substantial reductions in A, and vice versa. In cases where a region's reduction in MCPI is primarily achieved by assisting the marginally poor in crossing the poverty line, with little impact on those who were in severe poverty, the improvement is reflected in H but not in A. Conversely, if a region's reduction in MCPI is primarily achieved by reducing the severity of poverty among those who remain poor, the improvement may not be reflected in H but can be observed through a reduction in A. Therefore, by examining changes in both H and A alongside the MCPI, valuable policy insights can be gleaned.

**Table 2:** H, A and MCPI Status across population subgroups in India among 0–4 Years Children

	**NFHS-4, 2015–16**							**NFHS-5, 2019–21**						
	**Pop Share**	**H**		**A**		**MCPI**		**Pop Share**	**H**		**A**		**MCPI**	
	%	%	s.e	%	s.e	est	s.e	%	%	s.e	%	s.e	est	s.e
**India**		46.6	0.003	51.7	0.001	0.241	0.002		27.4	0.002	46.2	0.001	0.126	0.001
**Place of residence**														
Urban	0.276	18.3	0.003	46.9	0.002	0.086	0.001	0.261	9.6	0.002	43.9	0.002	0.042	0.001
Rural	0.724	57.4	0.002	52.3	0.001	0.3	0.001	0.739	33.6	0.002	46.4	0.001	0.156	0.001
**Castes**														
Others	0.237	31.2	0.003	49	0.001	0.153	0.002	0.235	17.7	0.003	44.6	0.001	0.079	0.001
SC	0.221	52.5	0.004	51.7	0.001	0.272	0.002	0.238	32.4	0.003	46.5	0.001	0.151	0.001
ST	0.104	69.2	0.004	54.7	0.001	0.379	0.002	0.105	45.2	0.004	47.9	0.001	0.217	0.002
OBC	0.437	46.5	0.002	51.6	0.001	0.24	0.001	0.423	25.5	0.002	45.9	0.001	0.117	0.001
**Religions**														
Hindu	0.783	46.9	0.002	51.7	0.001	0.242	0.001	0.791	27.3	0.002	46.3	0.001	0.126	0.001
Muslim	0.168	50	0.004	52.1	0.001	0.261	0.002	0.163	30.1	0.004	45.8	0.001	0.138	0.002
Christian	0.021	33.6	0.009	50.1	0.003	0.168	0.004	0.022	21.9	0.006	47.3	0.003	0.104	0.003
Others	0.028	26.6	0.008	50.3	0.004	0.134	0.004	0.023	16.5	0.006	47.2	0.004	0.078	0.003
**Regions**														
North	13.4	34.9	0.003	51.3	0.001	0.179	0.002	13.6	17	0.002	44.3	0.001	0.075	0.001
West	12	27.2	0.005	46.9	0.002	0.127	0.002	12.5	16.5	0.004	45.1	0.002	0.074	0.002
Central	27.3	61.5	0.002	52.8	0.001	0.325	0.001	27.3	34.2	0.003	45.8	0.001	0.157	0.001
East	26.3	63.6	0.003	53.3	0.001	0.339	0.002	26.3	41.9	0.003	47.6	0.001	0.199	0.002
North-east	3.7	49.1	0.004	49.5	0.001	0.243	0.002	4	31.5	0.004	45.6	0.002	0.144	0.002
South	17.3	19.2	0.003	45	0.002	0.087	0.002	16.4	8.6	0.002	43.4	0.002	0.037	0.001

*Source*: Author's calculation based on NFHS 2015–16 & 2019–21

**Fig. 1:**
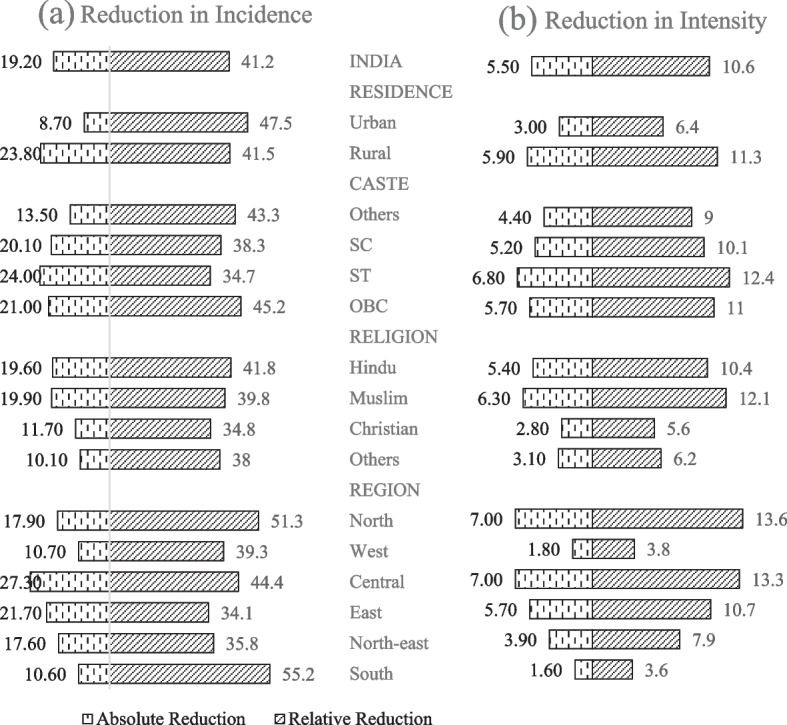
Changes in (**a**) Incidence and (**b**) Intensity of child poverty across population subgroups between 2015–16 and 2019–21

India's performance in terms of MCP reduction across places of residence (rural and urban) and across the geographical region is illustrated in Table 2 and Fig. 1(a) & (b). Notably, both rural and urban areas have experienced decreases in MCPI, H and A, although the extent of these reductions varies. Absolute reduction of incidence of MCP (H) was higher in rural areas compared to the urban areas, but in case of relative reduction, the opposite was true. However, in terms of intensity of MCP (A), rural areas marked a higher reduction than urban areas in both absolute and relative terms. While looking at the changes across the six regions of India, reduction in all the MCP subindices was observed, though the magnitude of reduction varied significantly across the regions. The incidence of MCP were highest in the Eastern region, followed by the Central and Northeastern regions in both the time periods. The Southern region continues to have the least MCPI value, with a score of 0.037. The absolute reduction of H and A were highest in the Central and Eastern regions. Whereas, the relative reduction of H was highest in the Southern region followed by the Northern region, and the reduction in A was highest in the Northern and Central regions. The relative reduction in MCPI was largest for Northern regions, followed by Southern regions.

In the context of India, two particular social groups of interest are caste and religion, as highlighted in the study by Alkire & Seth, 2015 [38]. Looking across castes, it can be observed that there was a reduction in H, A, and MPCI for each of its four main subgroups. The most rapid decline in absolute poverty, as measured by both MCPI and H, was observed for the STs, while the slowest drop has occurred in the Others category. In terms of relative reduction, the highest reduction in both H and MCPI has been observed in the OBC category, i.e., 45.2% and 51.3%. The relative reduction of the intensity of MCP was highest in the ST category, with 12.4% reduction. It is worth noting that from the above-mentioned figures, OBC category children performed better in reducing the MCP during the period 2015–16 and 2019–21, followed by the STs and SCs category.

When examining the shifts in poverty levels across different religious groups, it becomes apparent that both the A and H indices, as well as the MCPI, have decreased across all religious subgroups. In terms of absolute reduction of incidence of MCP, both Muslims and Hindus recorded a comparable amount of reduction (19.9 vs 19.6 percentage points), but in terms of relative reduction, it was in favour of the Hindus: while the reduction in H for the Muslims was 39.8%, Hindus marked a reduction of 41.8%; but, in intensity of MCP, Muslims recorded the highest reduction in both absolute and relative terms. However, in overall, the relative reduction in both H and MCPI was highest in the case of Hindu community children. Through the subgroup analyses, it becomes evident that poverty reduction has generally occurred at a faster pace in the subgroups with higher poverty levels, while progress has been comparatively slower in the subgroups experiencing lower levels of poverty. While there was a substantial reduction in poverty at the national level, progress has not been evenly distributed among various subgroups. In fact, the disparity in poverty levels across these subgroups has actually increased between the periods 2015–16 and 2019–21.

#### Uncensored and censored headcount ratio

Table 3 illustrates the absolute change and relative change in uncensored headcount ratios, respectively. We observed changes in the rate of deprivations in every indicator across population subgroups and geographic regions between 2015–16 and 2019–21. The progress in each indicator varies greatly across population subgroups and geographic locations. The table indicates India registered its highest reduction in the indicators electricity, followed by birth registration and assisted delivery, with a reduction of 73.4%, 47.1% and 44%, respectively. The same pattern follows across the geographic locations and population subgroups. It is worth noting that in all indicators, the biggest improvements were recorded in rural areas as compared to urban areas. Across the six regions, while reduction was observed in most of the deprivation indicators, the prevalence of stunting has increased in the Northeast, South, and West; and the prevalence of wasting have increased in the Northeastern region. It is also noteworthy that, the Northeastern region performed poorly in terms of reducing the rate of deprivation in drinking water and housing condition compared to all other regions of the country. Similarly, the Western region fared worse than all other regions in the hand hygiene indicator.

**Table 3:** Absolute and Relative change in uncensored headcount ratio by indicator between 2015/16 and 2019–21 across population Subgroups and geographic locations in India

	**Absolute Change (in percentage points)**														**Relative Change (in percentage)**													
	**ST**	**WT**	**AD**	**IMZ**	**ME**	**BR**	**CF**	**EC**	**HC**	**OC**	**INF**	**DW**	**SN**	**HH**	**ST**	**WT**	**AD**	**IMZ**	**ME**	**BR**	**CF**	**EC**	**HC**	**OC**	**INF**	**DW**	**SN**	**HH**
**India**	**-2.7**	**-1.9**	**-8.1**	**-7.3**	**-9.1**	**-9.4**	**-15.3**	**-11.6**	**-4.7**	**-7.7**	**-12.7**	**-2.5**	**-23.6**	**-9.6**	**-6.9**	**-9.2**	**-43.9**	**-22**	**-30**	**-47.1**	**-23.7**	**-73.4**	**-8.9**	**-13.7**	**-33**	**-30.5**	**-40.3**	**-29.4**
**Place of residence**																												
Urban	-0.8	-1.6	-4.1	-6.1	-4.6	-4.5	-9.5	-2	-1.5	-7.4	-5.6	-1.2	-12.4	-6.3	-2.4	-8	-41.2	-20	-28.3	-41.1	-40.8	-62	-7.2	-14.3	-39.5	-35.3	-35	-32.3
Rural	-3.6	-2.1	-9.8	-7.8	-11.1	-11.4	-18.9	-15.5	-7.1	-7.5	-17	-3.4	-28.1	-11.4	-8.6	-9.6	-45	-22.8	-31	-48.7	-23.3	-75.9	-11	-13	-36.1	-33.2	-40.7	-29.9
**Castes**																												
Others	-0.9	-1.4	-6.9	-6.6	-5.9	-6.1	-12.7	-7.3	-3.3	-8.2	-10	-1.7	-16.2	-4.2	-2.7	-7.4	-46.1	-19.7	-31.1	-46.2	-24.5	-74.6	-8.1	-16.7	-34.3	-27.4	-36.7	-15.9
SC	-3.3	-1.8	-7.6	-6.5	-9.6	-8.8	-18.2	-13.4	-4.8	-6	-12.8	-2.2	-26.4	-12.5	-7.7	-8.3	-40	-20	-28	-42.7	-25.3	-72.4	-8.1	-9.7	-32.1	-32.1	-38.3	-32.4
ST	-2.4	-4.1	-12.7	-11.4	-11	-12.3	-14	-13	-5.9	-9.1	-16.6	-7.6	-29.1	-15.1	-5.4	-15.2	-45.4	-31.9	-25.2	-51.4	-16.1	-67.9	-7.9	-15.1	-29.9	-34.6	-37.3	-29.4
OBC	-3.6	-1.8	-8.1	-7.2	-10.4	-10.9	-16.5	-13	-6.2	-7.6	-15.3	-2.3	-25	-10.6	-9.3	-8.8	-44.8	-21.6	-32.8	-48.6	-25.8	-78.5	-12.2	-13.3	-40.4	-34	-42.3	-35.3
**Religions**																												
Hindu	-2.8	-2.4	-7.2	-7.2	-9	-9.4	-16.1	-12.1	-5.5	-6.9	-13.3	-2.9	-24.9	-10.9	-7.2	-11	-42.5	-22.1	-30.3	-46.7	-24.1	-74.9	-10.2	-12.3	-36	-34	-39.9	-32.1
Muslim	-2.8	0.5	-13.4	-9.2	-11.6	-11.5	-13.9	-12.5	-3.9	-10.5	-16.8	-1.5	-19.5	-5.4	-7	2.8	-50.6	-23.6	-30	-51.5	-22.4	-77.2	-7.6	-16.9	-35.2	-29.1	-36.7	-18.2
Christian	1.4	-2.6	-4.1	-7.1	-2.1	-5.1	-12.2	-3.6	-3.5	-2.1	-5.7	-5.7	-16.7	-8.2	4.7	-13.6	-21	-21.9	-12.6	-36.2	-22.2	-47.3	-8.4	-5.1	-20.3	-32.3	-40.7	-22.7
Others	-2.9	-4.7	-5.2	0.8	-3.7	-1.9	-16	-3.8	-0.2	-8.1	-7.2	-0.7	-16.4	-7	-8.8	-22.7	-42.5	3.7	-21.6	-25.8	-29.7	-56.8	-0.4	-16.7	-33.1	-8.7	-40.1	-26.7
**Regions**																												
North	-4.6	-5.4	-8.1	-9.1	-9.4	-13.5	-10	-4.5	3.4	-8.8	-12.2	-3.1	-17.2	-12.3	-13.4	-27	-59.2	-31.2	-30.5	-69	-17.1	-77.4	10	-17.5	-50.7	-34.9	-38.6	-50.4
West	1.4	-0.6	-3.5	-4.2	-3.1	-1	-18.6	-3.5	-3.8	-7	-8.6	-2.1	-18.7	-2.2	4	-2.4	-35.3	-11.9	-20.8	-25.7	-39.6	-57.3	-12.9	-10.8	-37.4	-23.3	-37.8	-8.6
Central	-5.7	-2.7	-13	-7.7	-13.1	-15.2	-15	-14.7	-7.9	-10.4	-19.1	-3.5	-32.8	-9.6	-12.7	-13.2	-48.5	-19.8	-33	-49	-20.2	-65.9	-11.2	-15.9	-40.1	-42.3	-46.9	-38.4
East	-3.2	-0.1	-9.7	-7.6	-11.2	-9.8	-16.6	-23.2	-8.8	-5.6	-18.2	-2.5	-22	-10.6	-7.6	-0.6	-39.8	-23.3	-27.2	-38.3	-19.5	-86.1	-12.6	-10.4	-30.9	-33.2	-30.5	-23.9
North-east	0.5	3.4	-11	-7.9	-6.2	-3.5	-15.6	-13.1	-4.5	-4.9	-12.8	-3.1	-18.3	-8.3	1.5	21.8	-39.7	-17.7	-28.9	-35.4	-20.2	-62.2	-5.9	-16	-25.3	-15.9	-35	-20.6
South	0.6	-3.1	-0.9	-7.6	-4.7	-3.5	-18.4	-0.6	-2.8	-5.2	-2.9	-2	-20.8	-12.4	1.9	-15.5	-21.5	-30.2	-37.5	-47.4	-49.7	-60.1	-11.7	-10.3	-28.8	-33.4	-45.5	-31.5

*Source*: Authors' Calculation from NFHS, 2015–16 & 2019–21

*WT* Wasting; *ST* stunting, *AD* Assisted Delivery, *ME* Mother's education, *Inf* Availability of TV/Radio, *IMZ* Immunization, *BR* Birth Registration, *CF* Cooking Fuel, *EC* Electricity, *HC* Housing Condition, *OC* Over-crowding, *DW* Drinking Water, *SAT* Sanitation, *HH* Hand Hygiene

Among the castes, STs made a remarkable progress in reducing wasting, and increasing the coverage of birth registration and immunisation. Likewise, OBCs made remarkable advancements in electricity and housing condition. Among the religious groups, Muslims showed poor performance in nutritional status indicator, i.e., in wasting; similarly, Christians in assisted delivery; and Others religious group in immunisation, housing condition and drinking water.

To specifically address the wellbeing of disadvantaged children, we analyse the changes in censored headcount ratios. Table 4 presents all indicators' absolute and relative changes in the censored headcount ratios across the population subgroups. By focusing specifically on impoverished children, we observe that all population subgroups and geographic areas have managed to reduce the censored headcount ratios across all indicators. The percentage of children who were multidimensionally poor and lives in a household that is deprived of electricity have reduced by at least three-fourths between 2015–16 to 2019–21. Similarly, the relative reduction in censored headcount ratio in birth registration, sanitation facility and assisted delivery were also noticeably higher: 60.79%, 54.01%, and 53.8%, respectively. Across the places of residence, castes, religions, and regions, relative reduction in censored headcount ratio of indicators like electricity, birth registration, drinking water, assisted delivery, sanitation and cooking fuel were. It is intriguing to observe and monitor the alterations in all the pertinent indicators and recognise that none of the 14 MCP indicators remained unaltered throughout the analysed period.

**Table 4:** Absolute and Relative change in censored headcount ratio by indicator between 2015/16 and 2019–21 across population subgroups and geographic locations in India

	**Absolute Change (in percentage points)**													
	**ST**	**WT**	**AD**	**IMZ**	**ME**	**BR**	**CF**	**EC**	**HC**	**OC**	**INF**	**DW**	**SN**	**HH**
**India**	-9.71	-5.08	-9.73	-9.88	-13.00	-11.35	-20.97	-10.61	-16.84	-14.11	-15.96	-4.15	-22.61	-11.44
**Place of residence**														
Urban	-4.6	-2.25	-3.59	-4.73	-6.31	-4.07	-6.69	-1.68	-4.78	-7.41	-4.88	-0.95	-7.39	-3.68
Rural	-11.78	-5.82	-10.57	-10.8	-15.42	-12.54	-25.64	-15.15	-20.01	-16.86	-19.98	-4.01	-27.22	-13.24
**Castes**														
Others	-6.03	-2.9	-6.73	-6.99	-7.94	-5.98	-13.1	-6.79	-10.56	-9.42	-10.81	-1.88	-13.02	-5.87
SC	-10.84	-4.67	-7.95	-8.52	-13.27	-9.91	-22.04	-13.14	-16.13	-15.03	-15.25	-2.63	-24.2	-12.28
ST	-10.59	-8.82	-13.47	-12.45	-15.92	-13.01	-26.13	-12.91	-21.21	-17.29	-20.98	-8.65	-30.98	-19.25
OBC	-10.87	-4.92	-8.77	-9.6	-14.43	-11.85	-21.66	-12.69	-16.73	-15.46	-17.22	-2.74	-22.71	-10.08
**Religions**														
Hindu	-9.76	-5.09	-7.78	-8.84	-12.48	-10.12	-20.69	-11.71	-15.9	-14.1	-15.25	-3.41	-22.44	-11.1
Muslim	-10.51	-3.64	-13.33	-10.97	-16.39	-12.01	-20.02	-12.14	-15.61	-15.71	-19.53	-1.66	-19.93	-8.14
Christian	-2.94	-2.69	-4.38	-4.97	-3.45	-5.85	-10.3	-3.72	-8.67	-7.04	-7.05	-5.73	-13.57	-8.21
Others	-5.1	-3.45	-4.83	-4.36	-4.18	-2.21	-10.87	-3.48	-8.21	-7.02	-7.18	-1.78	-11.27	-7.48
**Regions **														
North	-9.13	-5.81	-6.91	-8.63	-13.36	-12.15	-17.07	-4.53	-9.32	-13.86	-12.84	-3.86	-16.13	-11.16
West	-4.98	-3.3	-3.43	-5.58	-4.82	-1.36	-11.36	-2.8	-6.58	-9.49	-6.95	-2.67	-11.3	-5.51
Central	-14.24	-6.35	-14.13	-14.22	-18.17	-16.83	-27.48	-14.9	-24.01	-21.47	-22.25	-3.99	-32.28	-11.25
East	-10.77	-4.62	-10.48	-8.8	-15.68	-11.35	-25.12	-22.17	-20.71	-13.19	-22.21	-3.04	-25.26	-13.85
North-east	-6.55	-1.49	-11.1	-9.89	-8.23	-4.25	-18.12	-12.54	-15.81	-7.57	-15.27	-3.31	-17.68	-9.94
South	-4.97	-3.46	-1.28	-3.71	-5.7	-3.36	-9.9	-0.55	-5.28	-8.37	-3.44	-1.71	-11.07	-7.22

	**Relative Change (in percentage)**													
	**ST**	**WT**	**AD**	**IMZ**	**ME**	**BR**	**CF**	**EC**	**HC**	**OC**	**INF**	**DW**	**SN**	**HH**
**India**	-38.32	-40.41	-53.82	-46.69	-45.87	-60.79	-45.99	-75.52	-41.00	-41.82	-48.06	-45.21	-54.01	-46.89
**Place of residence**														
Urban	-45.73	-43.28	-59.36	-54.85	-54.4	-60.22	-56.85	-66.7	-46.59	-49.53	-56.2	-60.49	-51.81	-46.34
Rural	-38.5	-39.24	-55	-44.98	-45.47	-59.01	-46.79	-78.19	-40.87	-41.01	-48.97	-49.89	-52.9	-46.58
**Castes**														
Others	-37.92	-37.4	-58.59	-47.7	-49.52	-62.12	-47.79	-78.51	-42.35	-42.57	-50.27	-47.95	-50.64	-37.51
SC	-36.73	-34.37	-48.94	-39.7	-42.41	-53.02	-45	-74.36	-36.75	-37.87	-44.06	-50.14	-50.43	-46.48
ST	-29.81	-40.57	-52.43	-43.92	-37.8	-57.83	-38.77	-69.96	-34.97	-36.39	-41.81	-44.68	-48.54	-43.45
OBC	-43.52	-42.43	-58.16	-48.91	-49.91	-60.96	-51.02	-81.12	-44.77	-45.29	-53.95	-55.84	-55.7	-51.22
**Religions**														
Hindu	-38.76	-40.44	-54.08	-45.73	-45.74	-58.37	-47.36	-77.43	-40.9	-41.36	-48.62	-51.73	-52.62	-47.7
Muslim	-39.39	-32.14	-59.66	-45.85	-48.02	-61.86	-45.62	-79.5	-39.43	-41.89	-50.95	-42.86	-50.48	-37.77
Christian	-19.41	-33.19	-29.5	-32.75	-24.1	-49.49	-33.27	-52.55	-32.46	-36.51	-32.67	-45.31	-52.92	-39.28
Others	-35.51	-38.72	-51.21	-42.11	-32.46	-36.27	-43.38	-59.24	-36.03	-38.81	-40.86	-28.58	-49.6	-43.08
**Regions **														
North	-52.21	-60.68	-71.48	-59.48	-54.56	-78.05	-54.99	-80.93	-43.13	-52.71	-63.84	-55.16	-56.7	-62.75
West	-32.98	-33.84	-52.26	-42.84	-39.32	-47.21	-47.85	-61.25	-37.51	-40.44	-46.98	-44.87	-46.74	-34.09
Central	-42.04	-44.16	-60.23	-50.71	-48.97	-60.98	-48.72	-69.58	-43.84	-44.72	-52.34	-56.55	-59.19	-50.89
East	-31.81	-28.15	-47.43	-36.23	-39.52	-48.74	-40.8	-86.71	-36.86	-31.52	-43.8	-49.07	-43.83	-40.58
North-east	-28.03	-14.04	-48.01	-37.27	-41.23	-49.21	-38.51	-65.12	-33.94	-33.76	-38.84	-23.1	-47.65	-33.07
South	-49.29	-54.89	-51.35	-52.52	-58.09	-69.73	-66.59	-69.79	-50.38	-55.37	-55.6	-59.01	-62.79	-53.8

*Source*: Authors' Calculation from NFHS 2015–16 & 2019–21

*WT* Wasting, *ST* stunting, *AD* Assisted Delivery, *ME* Mother's education, *Inf* Availability of TV/Radio, *IMZ* Immunization, *BR* Birth Registration, *CF* Cooking Fuel, *EC* Electricity, *HC* Housing Condition, *OC* Over-crowding, *DW* Drinking Water, *SAT* Sanitation, *HH* Hand Hygiene

#### Shapley decomposition analysis

From Table 5 we see the Shapley decomposition analysis. To illustrate this, we have partitioned the analysis by the level of disaggregation as presented in Equation. We will first look at the overall contribution of each subgroup and then divide this effect into demographic and within-group effects. Finally, we will divide the within-group effect into incidence and intensity effects.

**Table 5:** Shapley decomposition of the changes in multidimensional child poverty across population subgroups and geographic locations in India

	**Place of residence**			**Caste**					**Religion**					**Region**						
	Urban	Rural	Total	Others	SC	ST	OBC	Total	Hindu	Muslim	Christian	Others	Total	North	West	Central	East	Northeast	South	Total
**Multidimensional child poverty**																				
**2015–16**																				
H	18.3	57.4	46.6	31.2	52.5	69.2	46.5	46.6	46.9	50	33.6	26.6	46.6	34.9	27.2	61.5	63.6	49.1	19.2	46.6
A	46.9	52.3	51.7	49	51.7	54.7	51.6	51.7	51.7	52.1	50.1	50.3	51.7	51.3	46.9	52.8	53.3	49.5	45	51.7
MCPI (%)	8.6	30	24.1	15.3	27.2	37.9	24	24.1	24.2	26.1	16.8	13.4	24.1	17.9	12.7	32.5	33.9	24.3	8.7	24.1
% Share Population	27.6	72.4	100	23.7	22.1	10.4	43.7	100	78.3	16.8	2.1	2.8	100	13.4	12	27.3	26.3	3.7	17.3	100
% MD-poor	10.81	89.19	100	15.91	24.96	15.45	43.68	100	78.85	18.06	1.48	1.62	100	10.06	6.97	36.09	35.84	3.92	7.13	100
**2019–21**																				
H	9.6	33.6	27.4	17.7	32.4	45.2	25.5	27.4	27.3	30.1	21.9	16.5	27.4	17	16.5	34.2	41.9	31.5	8.6	27.4
A	43.9	46.4	46.2	44.6	46.5	47.9	45.9	46.2	46.3	45.8	47.3	47.2	46.2	44.3	45.1	45.8	47.6	45.6	43.4	46.2
MCPI (%)	4.2	15.6	12.6	7.9	15.1	21.7	11.7	12.6	12.6	13.8	10.4	7.8	12.6	7.5	7.4	15.7	19.9	14.4	3.7	12.6
% Share Population	26.1	73.9	100	23.5	23.8	10.5	42.3	100	79.1	16.3	2.2	2.3	100	13.6	12.5	27.3	26.3	4	16.4	100
% MD-poor	9.15	90.85	100	15.15	28.13	17.3	39.42	100	78.85	17.96	1.78	1.41	100	8.46	7.54	34.11	40.21	4.55	5.13	100
**Decomposition**																				
Total % Contribution (M_0_ = 100 for India)	11.14	88.87	100.01	15.54	21.23	14.6	48.63	100	78.53	18.67	1.09	1.71	100	12.02	5.22	39.99	32.11	2.82	7.83	99.99
Demographic effect	0.84	-2.98	-2.15	0.2	-3.16	-0.26	2.19	-1.02	-1.29	0.87	-0.12	0.46	-0.07	-0.22	-0.44	0	0	-0.51	0.49	-0.68
Within group effect	10.3	91.85	102.15	15.33	24.38	14.86	46.44	101.02	79.82	17.8	1.2	1.25	100.07	12.24	5.66	39.99	32.11	3.32	7.35	100.67
Incidence of poverty effect (H) (%)	9.31	74.75	84.06	13.1	19.92	11.29	38.68	82.99	66.04	14.14	1.06	1.1	82.34	10.1	5.24	32.02	25.19	2.8	7.01	82.36
Intensity of poverty effect (A) (%)	0.98	17.08	18.06	2.23	4.45	3.56	7.75	17.99	13.77	3.66	0.14	0.15	17.72	2.14	0.42	7.97	6.92	0.53	0.33	18.31
**Health effect** **(in reducing intensity) (%)**	**0.21**	**2.87**	**3.08**	**0.43**	**0.73**	**0.62**	**1.32**	**3.1**	**2.31**	**0.7**	**0.02**	**0.03**	**3.06**	**0.39**	**0.08**	**1.44**	**1.05**	**0.1**	**0.06**	**3.12**
Stunting	0.06	0.87	0.93	0.11	0.25	0.15	0.42	0.92	0.72	0.19	0	0.01	0.92	0.12	0.02	0.42	0.33	0.02	0.02	0.93
Wasting	0.03	0.43	0.46	0.05	0.11	0.12	0.19	0.47	0.37	0.07	0	0.01	0.45	0.07	0.02	0.19	0.14	0.01	0.01	0.44
Assisted Delivery	0.05	0.78	0.83	0.13	0.18	0.18	0.34	0.83	0.57	0.24	0.01	0.01	0.83	0.09	0.02	0.42	0.32	0.04	0.01	0.88
Immunisation	0.06	0.79	0.86	0.13	0.19	0.17	0.37	0.87	0.65	0.2	0.01	0.01	0.86	0.11	0.03	0.42	0.27	0.03	0.02	0.87
**ECD effect** **(in reducing A) (%)**	**0.28**	**4.11**	**4.39**	**0.52**	**1.06**	**0.79**	**2.03**	**4.4**	**3.31**	**1.03**	**0.03**	**0.02**	**4.39**	**0.65**	**0.06**	**2.06**	**2.51**	**0.19**	**0.09**	**5.56**
Mother education	0.17	2.27	2.44	0.3	0.6	0.44	1.11	2.45	1.83	0.6	0.01	0.01	2.45	0.34	0.05	1.07	0.95	0.06	0.05	2.51
Birth registration	0.11	1.84	1.96	0.22	0.45	0.36	0.91	1.95	1.48	0.44	0.02	0.01	1.94	0.31	0.01	0.99	0.69	0.03	0.03	2.06
**Standard of living effect** **(in reducing A) (%)**	**0.28**	**5.75**	**6.03**	**0.76**	**1.49**	**1.08**	**2.58**	**5.91**	**4.55**	**1.21**	**0.04**	**0.05**	**5.85**	**0.58**	**0.15**	**2.6**	**2.51**	**0.19**	**0.09**	**6.12**
Cooking fuel	0.07	1.51	1.58	0.2	0.4	0.29	0.67	1.55	1.21	0.29	0.01	0.01	1.53	0.17	0.04	0.65	0.61	0.05	0.03	1.56
Electricity	0.02	0.89	0.91	0.1	0.24	0.14	0.39	0.87	0.69	0.18	0	0	0.87	0.05	0.01	0.35	0.54	0.03	0	0.98
Housing	0.05	1.18	1.23	0.16	0.29	0.23	0.52	1.2	0.93	0.23	0.01	0.01	1.18	0.09	0.03	0.57	0.5	0.04	0.02	1.25
Over crowding	0.08	0.99	1.07	0.14	0.27	0.19	0.48	1.08	0.83	0.23	0.01	0.01	1.07	0.14	0.04	0.51	0.32	0.02	0.03	1.05
Information	0.05	1.18	1.23	0.16	0.28	0.23	0.53	1.2	0.89	0.28	0.01	0.01	1.2	0.13	0.03	0.52	0.54	0.04	0.01	1.28
**WASH effect** **(in reducing A) (%)**	**0.22**	**4.36**	**4.58**	**0.52**	**1.19**	**1.08**	**1.83**	**4.62**	**3.61**	**0.72**	**0.05**	**0.05**	**4.43**	**0.53**	**0.13**	**1.87**	**1.71**	**0.14**	**0.11**	**4.49**
Drinking water	0.02	0.39	0.41	0.05	0.08	0.16	0.14	0.43	0.33	0.04	0.01	0	0.39	0.07	0.02	0.16	0.12	0.02	0.01	0.39
Sanitation	0.13	2.67	2.8	0.33	0.73	0.57	1.17	2.79	2.19	0.48	0.03	0.03	2.73	0.27	0.07	1.27	1.02	0.08	0.06	2.78
Hand hygiene	0.07	1.3	1.37	0.15	0.37	0.35	0.52	1.39	1.08	0.2	0.02	0.02	1.31	0.19	0.04	0.44	0.56	0.05	0.04	1.31

*Source*: Author's own calculation based on NFHS 2015–16 & 2019–21

The current concern revolves around determining the extent to which different sub-groups have contributed to reducing MCP in India. The contribution of each sub-group to the overall reduction in child poverty is assessed based on factors such as the initial poverty level, rate of poverty reduction and the population growth within each category. At the bottom of Table 5, the Shapley decomposition results display the contributions of various factors such as place of residence, caste, religion, and region to the overall poverty reduction among children aged 0–4 years in India. These contributions are further analysed at a primary level, considering the within-group and demographic effects. The demographic effect demonstrates variations in poverty resulting from changes in population share due to differences in population growth rate compared to other divisions, which could be attributed to migration dynamics or disparities in mortality and birth rates [32, 38, 39]. From a policy perspective, examining the marginal impact of poverty reduction within each specific subgroup is crucial, which is captured by the within-group effect. The study uncovers intriguing findings.

Across the places of residence, rural areas children have a higher contribution to overall poverty reduction in India of 88.87%, a higher percentage than its population share and a quite lower percentage than its multidimensional poverty share. We see that among the caste OBC category, children fare the best, as this sub-group contributes 48.6% of the total change in multidimensional child poverty, but it contributed more than its population share, and the same pattern has been shown in the Muslim sub-group. Across the religion, Hindu children contribute significantly higher (78.53%) to overall poverty reduction in India, but in fact, it contributed an equivalent percentage to its population share and multidimensional poverty share. Muslim sub-group also contributed more (18.67%) to the overall reduction of poverty than its population share. Central, followed by the Eastern regions, have a high contribution to overall poverty reduction, but they contributed more than their population shares. East region and ST category children did not reduce the percentage of multidimensional poor. In contrast, the rest of the sub-groups of children contributed less than their population shares.

Further, we decompose the total contribution of each group in their demographic and within-group effects. We can observe that in all the subgroups, most of the changes in MCP are driven by the within-group effect. For rural children, SC and ST, Hindu and Christian, and North, West and North-east children, the demographic effect barely decreased the change in poverty. On a second level of disaggregation, we may determine whether the change in the within-group effect was due to a change in the incidence of poverty or a change in the intensity of poverty among poor children by sub-group. The results show that the biggest change was due to the incidence of poverty, the overall effect of which accounts for nearly 83 percent of the change in M_0_. In all the subgroups, the changes in incidence are much higher than the intensity effect. The present study reveals that the subgroup classifications took two distinct approaches to poverty reduction, either by lowering the incidence of poverty or the severity of deprivation among the poor.

Now the question arises of how the profile of deprivation among multidimensionally poor children has changed. One of the favourable aspects provided by the adjusted headcount ratio is that, after the identification step, it can be broken down by dimension. This attribute proves particularly useful when examining changes over time, as it helps in understanding the linkages between variations in dimension and overall poverty changes. By analysing the changes in uncensored/raw and censored headcount ratios, as done in the previous section, we gained valuable insights into which dimensions and indicators were responsible for driving the overall change. In the Shapley decomposition (Eq. 7), we take a step further and calculate the contribution of each dimension and indicator to the changes in the MCPI by reducing the intensity of deprivations among poor children (see Table 5). Finally, we break down the intensity effect to study the marginal effect of each indicator. The main dimension that is driving the improvement in the intensity effect is the standard of living, followed by WASH, ECD and health. It means that poor children are considerably less deprived of their standard of living by 2019–21 in comparison to how they were in 2015–16. This decomposition shows us how the deprivation profile among poor children is changing in India.

The analysis by population subgroups and geographical locations provides further insights. Rural areas have experienced the greatest improvements in alleviating deprivation concerning mothers’ education and sanitation for poor children. In terms of the intensity of poverty reduction, all four dimensions in rural areas have shown progress compared to urban areas. While in urban areas, all dimension has a somewhat similar impact on changes in poverty intensity, with minor variations. When considering the castes, the greatest improvements in reducing poverty intensity have been observed in the standard of living and ECD dimensions. Children belonging to the OBC category help reduce poverty's intensity effect in all dimensions compared to all other castes. Similarly, among various religious groups, Hindu children play a crucial role in reducing the intensity effect of poverty in all dimensions compared to children from all other religions. Likewise, across different regions, Central and East region children help in reducing the intensity effect of poverty in all the dimensions compared to children from all other regions.

## Discussion

This paper has measured the changes in MCP among 0-4 years children across population subgroups (i.e. castes and religions) and geographic locations (places of residence and regions) of India by using the two rounds of the Indian Demographic Health Survey (also known as National Family Health Survey) conducted in 2015–16 and 2019–21. The MCPI presented in this study includes indicators specific to children's development and future wellbeing, which was not explored in the national MPI (NITI Aayog, 2021). Fourteen indicators that were considered for the construction of MCPI represented the nation's SDGs targets and children's rights throughout the four dimensions (health, ECD, standard of living, and WASH). This study contributes to a better understanding of child poverty in the country by including indicators of child deprivations that align with the SDGs targets.

In this study, we analysed the pattern of reduction in MCPI, H, A, censored and uncensored headcount ratios across the population subgroups. Looking across castes, among 0–4 years children, we clearly see that there have been reductions in H, A and MCPI for each of the four main subgroups. The extent of poverty reduction, both in absolute and relative terms, varied among different caste groups and was not uniform. Here it is noted that the highest relative reduction of poverty in both 'H' and MCPI was observed for the OBCs, and the relative reduction in the A was highest in the ST category. From these findings, it is clear that children belonged to OBC category performed better during 2015–21; and the STs, followed by the SCs continued to remain as the poor performer, a scenario that is similar to the findings of previous studies [26, 37–39]. However, these above studies were based on household as the unit of analysis, while our study based on child aged 0-4 years as the unit of analysis. Based on these considerations, it can be inferred that caste disparities in India, specifically in relation to MCP levels, have not diminished. A substantial body of literature demonstrates that historically, as well as presently, Scheduled Castes and Scheduled Tribes have faced significant disadvantages across various indicators, such as poverty, material and social deprivation, and access to essential services and entitlements [39]. Because historically, these groups have faced discrimination, and thus exclusion, which refers to the process by which individuals or groups are prevented from participating fully in a society's economic, social, political and cultural life. Despite the fact that the government undertook several projects and programmes for the upliftment of these groups, which were backed by substantial financial support, their economic and social standing has remained largely unchanged [57].

Exploring the changes in poverty across religious groups, we find that both 'A' and 'H' and MCPI have declined among all major religious subgroups. Alkire et al. [39] also found a positive observation from religious disaggregation. They considered all religious groups halved their multidimensional household poverty. The relative reduction in both 'H' and MCPI was highest in the case of less poor communities, i.e. Hindu community children. Here it is noted that both absolute and relative reduction in the intensity of poverty was merely improved in Muslim children, and it is consistent with the study of Das et al. [26] in which they analysed the household MPI. Despite the huge progress, children belonged to Muslim category are still the poorest religious subgroup. According to Alkire et al. [39], in India Muslim household are still the poorest religious group, with almost every third of Muslim multidimensionally poor, compared to every sixth Christian and it coincides with the finding of another study conducted by John & Mutatkar [58]. A possible reason for this could be unequal economic development and differences in standard of living, consumption pattern, occupation structure, and endowment of physical and human capital between these groups [58]. Based on these findings, it can be inferred that disparities based on religion persist, as the degree of decline in multidimensional child poverty differs between the Muslim and Hindu populations in terms of both absolute and relative terms. These results validate the findings presented in the research conducted by Alkire & Seth [38].

In this study, we observed both rural and urban areas have experienced absolute reductions in MCPI, 'H' and 'A' with different magnitudes, and it is concurrent with the study of Alkire & Seth [38]. Absolute reduction of poverty was higher in rural areas than the urban areas, but in case of relative reduction, the opposite was true. Such findings confirm the work by Alkire et al., [39] but are opposite to the finding of Das et al.[26]. The only difference is that our study based on the child and their study based on household specific poverty. It indicates urban children performed better than rural children. It is worth noting that the rural intensity decreased by merely 11.3 percentage points in relative terms from 2015–16 to 2019–21, which is greater than the reduction in urban areas, and this result was consistent with the study of Alkire & Seth [38] and Alkire et al. [39]. This may lead to rural–urban migration, which slows down the apparent rate of poverty reduction in urban areas [38]. Since there is a difference in absolute and relative decline, we can assume that rural–urban disparity in MCPI continues, but the disparity has narrowed between 2015–16 and 2019–21. Here we may conclude that the government of India has implemented various policies and programmes in both rural and urban areas to improve the standard of living of poor people. Through this programme, many poor people engaged in various work and got benefitted. One such program, known as Deendayal Antyodaya Yojana–National Rural Livelihoods Mission (DAY-NRLM), was implemented with the objective of promoting various livelihood opportunities for impoverished rural households [59]. Similarly, similar initiatives were also introduced in urban areas, such as the Deendayal Antyodaya Yojana–National Urban Livelihoods Mission (DAY-NULM), which aims to provide credit facilities and skill development to the urban poor on a universal scale. In order to enhance the health conditions of both rural and urban populations and reduce infant and maternal mortality rates, the National Urban Health Mission (NUHM) and National Rural Health Mission (NRHM) were launched [59]. Through these programmes, skilled attendants support safe institutional delivery in both rural and urban areas. Incentive schemes such as Janni Suraksha Yojana (JSY) and Janani Shishu Suraksha Karyakaram (JSSK) have been instrumental in increasing mechanical deliveries, increasing antenatal care visits and reducing under-five mortality [59]. The evidence from DAY-NRLM and DAY-NULM indicates that these programs have the potential to enhance the incomes of both the rural and urban poor by providing them with diverse livelihood opportunities [59]. Such direction leads us to conclude that the condition of children living in rural and urban households has increased.

Similarly, we consider progress across six regions of India. H, A and MCPI were reduced over time across all regions, but the pattern of reduction widely varied across the regions. The absolute reduction of poverty incidence and intensity was highest in the Central and Eastern regions. Whereas the relative reduction of 'H' was highest in the Southern and Northern regions, and the relative reduction in intensity was highest in Northern and Central regions. The relative reduction in MCPI was highest in Northern region followed by Southern region. Based on these findings, we can argue that regional disparities persist in terms of reducing MCPI, H and A Despite India's rapid economic growth over the past few decades, there is considerable evidence of marked regional disparities in many spheres of socioeconomic development, infrastructure, education, healthcare and per capita income [60–63].

One of the leading causes of regional disparities in India is the uneven distribution of natural resources and infrastructure: some regions have more fertile land, better access to water, and more developed transportation networks than others [64]. This can lead to differences in agricultural productivity, industrial growth, and overall development. For example, the Southern region is better developed in infrastructure facilities as compared to other regions; however, it is agriculturally low-developed as compared to the Northern and Central regions [65]. Another factor contributing to regional disparities is historical and cultural differences. Some regions of India have historically been more affluent and have greater access to education and other opportunities. Government policies and programs have also played a role in exacerbating regional disparities. For example, development projects have often focused on the most developed regions of the country, neglecting the needs of more disadvantaged areas [65]. Understanding the factors that contribute to child poverty and deprivation, as well as their nature and magnitude, is crucial for equitable and inclusive development [2]. As regional level analysis plays a major role in determining inequitable access to socioeconomic resources and inequalities in the development indicators across the nation.

In India, the uncensored headcount ratio dropped between 2015–16 and 2019–21 for most indicators. The proportion of deprived children in several indicators, however, has not changed significantly over time, highlighting the areas in which there is still some room for improvement. These include overcrowding in SC children, hand hygiene in Other category castes, wasting in Muslim, assisted delivery in Christian, immunisation, housing condition and drinking water in Other category religions, stunting in North-east, West and South regions, wasting, housing condition and drinking water in North-east region and hand hygiene in Western region. Across geographic locations and population subgroups, the highest improvement is recorded in the context of electricity, birth registration and assisted delivery.

The censored headcount ratios decreased on all indicators across all the population subgroups and geographic locations listed above. Across places of residence, castes, religions, and regions the indicators like electricity, birth registration, drinking water, assisted delivery, sanitation and cooking fuel made significant improvements between 2015–16 to 2019–21. This may be the result of the programmes like "Deendayal Upadhyaya Gram Jyoti Yojana" (launched in 2014) that focuses on rural electrification, and "Pradhan Mantri Sahaj Bijli Har Ghar Yojana – Saubhagya" and "Ujwal Bharat" that aim to connect all unconnected households to electricity; And the "National Rural Drinking Water Programme" (launched in 2009) and "Jal Jeevan Mission" (launched in 2019) to provide piped water to every household in the country by 2024 [58]. The Government of India introduced the "Pradhan Mantri Ujjwala Yojana" in 2016 as part of its clean cooking fuel policy. Through this scheme, economically disadvantaged households were provided with liquid petroleum gas (LPG) cylinders and stoves [26]. This reduction in deprivation related to cooking fuel contributes to achieving SDG-7, which focuses on ensuring "access to clean, affordable, and modern energy for all". Similarly, the Government of India implemented various programs to address the deprivation in sanitation. The "Swachh Bharat Abhiyan," launched in 2014, aimed to create public demand for sanitation facilities. These initiatives align with SDG-6, which emphasises universal access to water and sanitation [47]. Additionally, efforts such as NRHM/NUHM have contributed to a decline in the percentage of individuals lacking access to health personnel during delivery. This progress supports India's advancement towards SDG-3, which aims to ensure "healthy lives and wellbeing for all at all ages" [47].

## Conclusion

This study proposes a consistent approach to analyse the factors that are driving the change of multidimensional child poverty among children aged 0–4 by using the Alkire and Foster, 2011 and Shapley, 2013 decomposition approach. The Shapley decomposition has the advantage of allowing us to identify significant effects of poverty intensity and incidence in MCPI reduction. We found that while the level of multidimensional child poverty has dropped, the overall picture is not that optimistic when all factors are included. The incidence effect was drastically reduced, whereas the intensity effect was marginal. The MCPI in India reflects the priorities set by national development programs aimed at reducing poverty. We argue that the trends observed in MCPI provide valuable insights into identifying children between 0–4 years who experience various forms of deprivation that cannot be solely captured by income-based measures of poverty. By utilising the Multidimensional Child Poverty measurement, it becomes possible to stimulate the development of a strategic action plan to address the persistent issues indicated by lagging indicators. We propose that the trends observed in MCPI can further fuel discussions regarding the evaluation of public policies in achieving development objectives while considering the overarching goal of sustainability. It allows us to keep track of SDG targets to reduce poverty by half of the population in all dimensions. Furthermore, trend analysis aids in monitoring the commitment to "Leave No One Behind". To that purpose, it is necessary to address the concerns of the vulnerable groups of children, e.g. Central and Eastern regions, the ST community, and the Muslim populations. This allows all members of Indian society to benefit from long-term development and prosperity.

## Data Availability

NFHS-4 and NFHS-5 data are available in the public domain at https://dhsprogram.com/data/available-datasets.cfm.

## Abbreviations

AF: Alkire & Foster
A: Intensity
ECD: Early Childhood Development
H: Headcount ratio
M_0_: Multidimensional adjusted headcount ratio
MCP: Multidimensional child poverty
MCPI: MCP Index, MPI: Multidimensional poverty index
NFHS: National Family Health Survey
OBCs: Other Backward Classes
SCs: Scheduled Castes
STs: Scheduled Tribes
SDGs: Sustainable Development Goals
WASH: Water sanitation and hygiene
UN: United Nation
